# Rhythmic training, literacy, and graphomotor skills in kindergarteners

**DOI:** 10.3389/fpsyg.2022.959534

**Published:** 2022-12-08

**Authors:** Aline Frey, Andrée Lessard, Isabelle Carchon, Joëlle Provasi, Loïc Pulido

**Affiliations:** ^1^Laboratoire de Neurosciences Cognitives, UMR 7291, CNRS – INSPE de l’Université d’Aix-Marseille, Marseille, France; ^2^Département des sciences de l’éducation, Université du Québec à Trois-Rivières, Trois-Rivières, QC, Canada; ^3^Centre de recherche interuniversitaire sur la formation et la profession enseignante (CRIFPE), Montréal, QC, Canada; ^4^Consortium régional de recherche en éducation (CRRE), Saguenay, QC, Canada; ^5^Observatoire interdisciplinaire de création et de recherche en musique, Université Laval (OICRM-ULaval), Québec, QC, Canada; ^6^Laboratoire CHArt, Cognitions Humaine et ARTificielle, EPHE - PSL, École Pratique des Hautes Études - Paris Sciences Lettres, Campus Condorcet, Aubervilliers, France; ^7^Consortium Régional de Recherche en Éducation (CRRE) et département des sciences de l’éducation, Université du Québec à Chicoutimi, Saguenay, QC, Canada; ^8^Centre de Recherche et d’Intervention sur la Réussite Scolaire (CRIRES), Université Laval, Québec, QC, Canada

**Keywords:** rhythm, literacy, spontaneous motor tempo, graphomotor abilities, kindergarten

## Abstract

**Introduction:**

The aim of this manuscript is twofold: first, to investigate the relationship between rhythmic, phonological and graphomotor skills in kindergarten children; and second, to evaluate the possible impact of rhythmic training on the two other skills.

**Methods:**

To that end, we selected a sample of 78 children in Québec. Forty-two children received rhythmic training (experimental group) and 34 arts training (active control group) during the same period (10 weeks). Before and after training, children in both groups were assessed for general skills (forward and backward memory span, vocabulary, non-verbal ability), rhythmic skills (synchronization and discrimination tasks), literacy skills (phonological skills - syllable counting, syllable deletion, rhyme discrimination – and invented spelling skills) and graphomotor skills (legibility of letter writing, quality of copying of geometric shapes).

**Results:**

Results showed correlations between the child’s rhythmic and literacy skills, as well as between rhythm synchronization and pen pressure. In addition, rhythmic training showed improvement in rhythmic abilities, but this did not transfer to literacy or graphomotor development (apart from a significant increase in the duration of pauses in both groups at post-test, with a larger improvement for the rhythm group).

**Discussion:**

These results are discussed in terms of duration and intensity of learning, and they highlight the possible benefits of informal rhythm practices in the classroom.

## Introduction

Rhythm, and especially the ability to be synchronized with one’s environment, is crucial for the sensory-motor, cognitive, emotional, and social development of young children (Feldman, 2007). Thus, children must be able to perceive the rhythms around them, produce rhythmic activities, and modify the rhythm of their activities in order to be in synchrony with the rhythm of their environment. The dynamic systems theory model (Jones, 1976; Jones and Boltz, 1989; Drake et al., 2000) explains how the subject’s selective attention works. Rhythmic sequences are broken down into different hierarchical levels of temporal regularity in relation to a preferred period of processing which is specific to each individual and acts as their reference period. The subject’s selective attention will spontaneously focus on events that occur during a period close to their reference period. On average, this reference period is 600 ms long in adults (Drake and Botte, 1993) and 400 ms long for children aged between 2 and 14 years (Provasi and Bobin-Bègue, 2003; Provasi et al., 2014). For children, this duration is accompanied by a time window of plus or minus 20% of its speed, meaning that they start to differentiate between two tempi once one is 20% faster or slower than the other. This 20% difference serves as the differentiation threshold between two tempi. It is thus more difficult for children to synchronize to a tempo of 600 ms +20% than to a tempo of 400 ms +20% (Provasi and Bobin-Bègue, 2003; Provasi et al., 2014). Similarly, in production, it is easier for children to synchronize to a 400 ms tempo than to a 600 ms tempo.

Furthermore, the ability to perceive and produce rhythmic patterns promotes fundamental human capacities that go beyond the strict rhythmic or musical field, including the coordination of action, and cognitive and language processing, specifically oral language skills (Tierney and Kraus, 2013). Thus, prosodic-level features such as speech rhythm and stress facilitate syllabic segmentation of the acoustic signal (Choi et al., 2005). Developmentally, speech rhythm is one of the first cues used by infants to segment the speech stream into words and word parts (Ramus et al., 2000) and parents naturally use emphatic stress and exaggerated rhythmic patterns when interacting with their infants.

Along those lines, a growing number of studies have examined the potential importance of rhythm perception and/or production in older children, between 2 and 5 years of age, and its relationship to reading skills. Working with American kindergarteners, Moritz et al. (2013) showed that performance in rhythm pattern copying marginally correlated to the ability to isolate initial phonemes, segment syllables, delete word and syllable parts from compound and multi-syllabic words, and segment sentences into words. In a beat synchronization task, Carr et al. (2014) demonstrated that American children between the ages of 3 and 4 who were unable to synchronize with an external auditory beat had poorer pre-literacy skills, specifically in phonological processing. More recently, Lundetræ and Thomson (2018) studied to what extent Norwegian first graders’ production of rhythm predicts reading and spelling difficulties. Significant group differences in emergent literacy skills and rhythmic tapping were found between children below and above the national threshold in word reading and spelling. More specifically, they were interested in whether students’ performance at the rhythm task at school entry served as a predictor of poor in word-reading and spelling abilities at the end of grade one. They found significant group differences in children’s ability to tap in time to an externally delivered beat, measured at school entry, when groups were defined upon whether children went on to score above or below the 20th percentile threshold in national assessment tests at the end of grade one. Taken together, these results demonstrate that rhythm production, perception and/or synchronization, and language skills are potentially interrelated and suggest the importance of temporal sensitivity for language learning in preschoolers. More specifically, it appears that rhythmic skills support speed naming of letters and phonological awareness (David et al., 2007), which are considered to be robust predictors of reading ability (Wolf and Bowers, 1999). Phonological awareness (PA) corresponds to the ability to manipulate speech sounds, phonemes, and syllables, and facilitates the decoding and the analysis of words during spelling (Bus and Van Ijzendoorn, 1999). This ability involves auditory processes for fine analysis of the temporal structure of sound patterns, which provides, for example, statistical clues to word boundaries (Culter, 2012). Phoneme distinctions may involve very fine temporal cues, such as distinguishing a /b/from a/p/ (Greenberg et al., 2006). Thus, as speech relies on fine temporal and rhythmic patterns, a body of evidence shows that rhythm mechanisms support PA. Rhythm skills could help children break down language chains into sublexical units, such as syllables, thus facilitating awareness of the phonological units of language (Goswami, 2011; Moritz et al., 2013).

Although much research has focused on the links between rhythmic and language abilities, much less has been devoted to the study of potential relationships between these rhythmic skills and handwriting skills. And yet, similarly to language and other complex human actions, handwriting is not just a succession of isolated acts, but rather an organized process, in which the time and the space of each motor unit (i.e., strokes, letters, and words) are contextually interdependent on one another within a larger unit (Van Galen and Teulings, 1983). Specifically, handwriting respects two principles based on rhythmic organization: the phenomena of homothety and isochrony (Lashley, 1951). Briefly, homotheticity states that the ratio between the durations of the motor events composing a writing movement remains unchanged even if the way in which the word is written is different (i.e., bigger, smaller, faster, or slower), and thus despite changes in overall duration. Isochrony, on the other hand, refers to the proportional relationship between the speed of execution of the movement and the length of its trajectory. In other words, depending on the size of the written output, the duration of execution of a movement remains stable; thus, writing speed adjusts proportionally to the size of the output. The typical example is a signature, which is always written at the same speed regardless of its size. In a study conducted on some 300 primary school children, Pagliarini et al. (2017) showed that these two principles of the rhythmic organization of handwriting are already present in children’s first handwriting productions, suggesting “that an internal representation of the rhythm of handwriting is available before the age in which handwriting is performed automatically” (p.1). These results suggest that, despite being a cultural acquisition, handwriting appears to be shaped by more general constraints on the temporal planning of movements and highlight the relationship with the rhythmic dimension that is present before handwriting movements become automatized (Evertz and Primus, 2017). Furthermore, Ben-Pazi et al. (2007) showed that rhythmic performance in a tapping task correlates with children’s handwriting quality. In this context, Véron-Delor et al. (2017) tested the hypothesis that rhythm can serve as an external cue that guides movement, through the natural and spontaneous tendency to synchronize with it (Bangert et al., 2006; Zatorre et al., 2007; Schaefer, 2014). In a child with severe writing difficulties, they show that playing rhythmic music in the background during a writing task significantly improved the duration, speed, and fluency of movement. However, apart from these few studies, and although it is known that there is a dynamic interaction between perception and action, both clearly involved in handwriting, the rhythmic dimension of writing has largely been neglected.

All of the above results are based on correlation analyses indicating links between rhythmic and literacy skills (language and/or handwriting skills). However, as correlation is not causality (Schellenberg, 2004; Chobert et al., 2014), one way to determine whether rhythm competence could mediate literacy skills is to conduct longitudinal studies that develop rhythmic skills and assess whether this development is accompanied by the development of literacy skills. Typically, in these kinds of studies, one group of children is trained in rhythmic skills, while the other group does something else, and the effect of these different practices on language and/or writing is measured before and after the training. Many studies have shown that musical training (i.e., instrumental practice, singing, pitch training) significantly improves language skills (e.g., pre-attentive perception, Frey et al., 2019; fluency, Zuk et al., 2014; novel word-learning and semantic associations, Dittinger et al., 2017) in children, showing beneficial transferrable effects of musical training. Of course, musical activities are usually based on rhythm, but training children solely on rhythmic aspects is less common. In Verney (2013)found that training in rhythmic activities led to increased PA, especially rhyme and syllable awareness. More recently, and in order to better understand the respective involvement of rhythm and pitch in the beneficial effects of musical learning on PA, Patscheke et al. (2018) randomly assigned preschoolers aged between 4 and 6 years old to a rhythm, pitch, or sport (control group) training group. The first two groups were trained three times a week during 20-min sessions over a period of 16 weeks. Results showed that only the pitch program improved PA abilities, even if the rhythm group also showed an increase which did not reach significance (when compared to the sports control group). Furthermore, rhythmic skills (as well as pitch perception) were not measured, which makes it impossible to know if the training worked well, and to know what the lack of improvement in language is related to.

Finally, this research also has educational and pedagogical implications. Indeed, most school-based learning is based on oral and written language skills, which must therefore be acquired as early as possible in education. Despite this, various surveys (e.g., PISA, OECD, 2019; PIRLS, Martin et al., 2017) have reported for several years that some children have early and persistent difficulties in processing language information and more specifically in identifying words all over the words, and notably for French speakers. These difficulties continue throughout schooling, showing that on average, 20% of adolescents have relatively significant reading difficulties in France for example. Thus, despite advances in scientific knowledge about learning psychology and pedagogy, teachers are still quite helpless to remedy students’ language difficulties (Frey and Sappey-Marinier, 2018). Faced with this observation, new alternatives appear indispensable, and an additional objective of this research is to propose a simple and playful approach for indirectly improving language and handwriting skills through rhythmic training.

Thus, the aim of this manuscript is twofold: first, to investigate the relationship between rhythmic, phonological, and graphomotor skills in 6-year-old children, and second, to evaluate the possible impact of rhythmic training on the other two skills. We hope to confirm correlations between rhythm and phonological skills and to observe understudied relationships between rhythm and handwriting skills. To achieve these objectives, we conducted a study on 78 children in Quebec. Rhythm skills were evaluated in both discrimination and synchronization tasks (Provasi et al., 2014). Phonological skills were classically assessed through syllable counting and deletion, rhyme discrimination, and initial phoneme identification tasks. We also set up an invented spelling task (Pulido and Morin, 2018), which is a way for children to explore the written code that involves variety of early literacy skills, such as alphabetic knowledge and phonological awareness in a more ecological way (Ouellette and Sénéchal, 2008). Finally, we examined the graphomotor component of handwriting (initiation and implementation of motor programs and neuromuscular execution) by evaluating the legibility of letter writing and their graphomotor characteristics, as well as the quality of the copying of geometrical shapes, on a digital tablet. These different skills were evaluated in pre- and post-tests, before and after 10 weeks of training, either in rhythm or in arts (active control group). We hypothesized that children trained in rhythm would not only improve their rhythmic skills, but also, through a transfer of learning effect, improve their literacy skills (phonological awareness and writing).

## Materials and methods

### Participants

Seventy-eight kindergartners from six classes across two schools in a middle town in Quebec, were involved in the experiment. Children whose teachers reported a learning disability were excluded from the analyses (2). Three classes were randomly assigned to the rhythm training (*n* = 42; 22 girls, mean age = 5 years 11 months, *SD* = 113 days), and the other three followed the arts training (*n* = 34; 17 girls, mean age = 5 years 10 months, *SD* = 129 days).

In Quebec, from the first year to 4 or 5 years of age, children mainly attend daycare centers where they are guided through playful activities to support their overall development (Ministère de la Famille, 2019). At age five, children can enter kindergarten, where they are offered informal or play-based activities. It aims to foster emerging literacy skills and children are expected to have phonological awareness skills and to be able to invent spelling by the end of kindergarten. None of them could read or write during the study.

### Measures

#### Control measures

In order to ensure the homogeneity of the two groups prior to trainings on skills that could influence the development of the skills we have focused on in this research (specifically phonological awareness and invented spelling), we carried out three control tasks during pre-testing.

To control for the vocabulary ability of children, we used the Expressive vocabulary task from CELF-CDN-F (Wiig et al., 2009). The task consists of naming 27 pictures for a total score of 54 (2 points for each spontaneous response and 1 point for a response given after a cue). The reported interitem reliability of this task is 0.90.

Raven’s Standard color Progressive Matrices (Raven et al., 1998) were used to control for the non-verbal ability of children. In this task, children are shown a picture with a partial mask, and have to decide which of six pictures completes the pattern. There are 36 items, and each correct is scored 1 point. The reported interitem reliability of this task is 0.76.

Finally, to control for working memory ability, we used both the forward and backward digit span from the Wechsler Intelligence scale for children (WISC IV, Wechsler, 2005), in which children have to repeat, either in the original order or backward, series of orally presented numbers (8 maximum in the forward test, 7 maximum in the backward test, 1 point for a correct response, total score out of 15).

#### Main measures

##### Rhythm skills

Children were given both discrimination and synchronization tasks (Provasi et al., 2014).

The two first discrimination tasks differed in the inter-stimulus interval (ISI) used in the rhythmic sequence of the reference period, that is 400 ms and 600 ms (400 ms is close to the reference period for children, and 600 ms is the reference period for adults). Children heard a first sequence of 15 beeps, followed by a second sequence of 15 more beeps 1,500 ms later. The children had to say orally whether the two sequences heard were the same or not.

Each pair of sequences contained a target sequence (with an ISI of 400 ms or 600 ms respectively), and the second sequence presented with the same ISI as the target sequence (i.e., 400 or 600 ms) for 4 trials, or 10, 20, 30 and 40% faster than the target sequence for 8 trials (2 of each). The position of the target sequence in each pair, the order of the 12 trials, and the order of presentation of these 2 discrimination tasks (400 or 600 ms) were counterbalanced between the children. Since children’s reference period is close to 400 ms, it is more difficult for them to discriminate two tempi around 600 ms than two tempi around 400 ms. As the threshold of discrimination of tempi is situated around 20%, the most difficult discrimination task is therefore that at 600 ms −20%, and the score from this task was considered the relevant discrimination score.

The motor rhythm task was divided into 3 successive phases: a synchronized tapping phase with an auditory tempo and two spontaneous motor tempo (SMT) phases, one just before and the other just after the synchronization phase. During the two phases of SMT, the children had to type on the spacebar of a computer keyboard, as regularly as possible, for a total of 30 successive strikes. During 11 trials of the synchronization phase, the children had to press the spacebar on a computer keyboard at the same time as a beep. Eleven trials differed in the duration of the ISI, either equal to their SMT (0%), or 10, 20, 30, or 40% faster or slower than their SMT, and two trials had an ISI of 400 and of 600 ms. The order of the 11 ISI trials was balanced between subjects. Synchronization to 400 ms ISI and 600 ms ISI were always the two last trials of synchronization phase. For these tasks, as in Provasi et al. (2014), we calculated the mean and standard deviation of Inter-Tap Interval (ITI) for the pre-, the post-synchronization phases, and for the synchronization phase. Because the ISI of the synchronization phase was dependent on the child’s SMT, the data were normalized by dividing the mean ITI by the mean ITI obtained during the pre-synchronization SMT. In addition, we calculated an adjusted value of the Rayleigh score as explained in Appendix 1 by Provasi et al. (2014). A higher adjusted Rayleigh score indicates a larger variability in rhythmic movement. The lower the value of the corrected Rayleigh, the more the taps are adjusted to the rhythmic stimulation. Since the children’s SMT is close to 400 ms, it is more difficult for them to synchronize at a 600 ms ISI than at a 400 ms ISI. A Rayleigh score corrected to 600 ms is thus the most relevant measure of synchronization when examining a rhythmic learning effect.

##### Literacy skills

Literacy skills of the participants were assessed through phonological awareness and invented spelling tasks. Four tasks of the phonological awareness subtest of CELF-CDN-F (Wiig et al., 2009) were used, consisting, respectively, in (i) counting the number of syllables in six words (syllable counting/6); (ii) repeating a word after removing a target syllable (syllable deletion/8); (iii) saying if two target words rhyme (rhyme discrimination/8) (iv); listening to words and identifying their first phoneme (phoneme identification/8). We chose these four tasks among the 11 proposed as Pulido and Morin (2018) did because they are close to the phonological mechanisms involved in word writing. The reported interitem reliability of this test is.96.

In the invented spelling task (Pulido and Morin, 2018), children were told to do their best to try to write four bisyllabic words with a simple spelling (in French: “ami,” “piano,” “citron,” “café”), with the letters they know. For each sound written with an appropriate grapheme, they earned one point, for a total maximum score of 17.

##### Graphomotor skills

To assess graphomotor skills, children were instructed to perform the following four tasks:

- Copy three geometrical shapes: a square, a circle, and a triangle.
- Copy three cycloids clockwise and three cycloids counterclockwise.
- Copy six letters presented in lower script: a, m, u, l, p, and e. These letters were chosen because of their variety in terms of forms.

For this copy task, a still model was presented to the children which disappeared when the pen touched the screen.

- Write of their first name.^1^

For each production, a legibility score was given following the agreement of two trained coders: 1 for a legible production and 0 for an illegible production, following the criterion presented in Table 1.

**Table 1:** Criterium used to assess the legibility of the participant’s productions in graphomotor tasks.

Task	Criteria
Copy of geometrical shapes	*Square*: 4 secant segments of equal length, opposite segments parallel; tolerance of a 2 mm opening in the shape *Circle*: closed shape with no angles *Triangle*: 3 secant segments. Tolerance of a 2 mm opening
Cycloids	*Clockwise cycloid*: three buckles made clockwise *Counterclockwise*: three buckles made counterclockwise
Letters and first name	For each letter, criteria of the Evaluation Tool of Children’s Handwriting (ETCH, Amundson, 1995)

These tasks were realized by writing or drawing on a digital tablet (Cintiq Creative Pen Display) connected to a Mac mini using Boot Camp. The Eye and Pen software (Alamargot et al., 2006) was used to present stimuli when necessary, and to record the graphomotor activity.

We thus collected different information about the kinematics of handwriting: (i) mean speed of the pen during production (in cm per second); (ii) mean pressure; (iii) number of pauses during production (threshold for the pause was set to 20 ms, considering the frequency of the digital tablet, *cf.* Alamargot and Morin (2015) for detailed explanations); and (iv) mean duration of pauses (in ms). For these four variables, we calculated an average value which concerns the drawing tasks (geometric and cycloid shapes) and the writing tasks (writing of isolated letters and writing of the first name).

#### Procedure

Each child was tested one time before the trainings and one time after. The tasks were performed face to face with one experimenter, in a quiet room, at school. The order of the tasks was: forward and backward digit, phonological awareness, Raven’s progressive matrices, expressive vocabulary (these four tasks were performed only in pre-testing), and rhythm and writing tasks. The whole experiment lasted about 30 min.

#### Trainings

Trainings were delivered to the children by their respective teachers, 5 min per day, 5 days per week, over a 10-week period, starting in February. This format was chosen to fit easily into students’ daily routines so as to minimize disruption and to encourage children’s involvement in the proposed activities (Harper and O’Brien, 2015). Although it is possible to observe improvements in cognitive abilities after music training in some studies, the frequency and duration of this training did not always influence the results among these studies (Sala and Gobet, 2020). However, in reading and writing, short, daily trainings are considered more effective than long, spaced-out interventions (Chapleau et al., 2020). Also, the effects of musical practice (neuroanatomical or functional changes) are observed when the practice is done a minimum of three times a week (Paquet, 2017).

Before the start of training, teachers received a half-day of instruction in which the interventions were explained to them, the tasks were demonstrated, and they practiced each task until they were completed correctly. During the 10-week training period, supervising researchers contacted all teachers once a week to ensure that there were no issues and to answer any questions. It was also ensured that both trainings involved the same level of child engagement. Finally, teachers were required to complete an intervention logbook, which ensured that the interventions were carried out accurately and involved the same level of child engagement in each condition.

##### Rhythm training

A typical rhythmic training session consisted of four different tasks (not necessarily in a fixed order), for a total of 5 min each day. The first task, “rhythmic vitamins,” consisted of repeating short rhythmic sequences using the fingers, hands, thighs, and feet (duration: 1 min). To do this, one-minute videos of pre-recorded sequences were projected to a screen in the classroom, visible to all children.

These pre-recorded sequences were produced by a researcher in the team, who holds a level II certification in the Orff Schulwerk approach (Orff Canada), using a progression adapted to the rhythmic abilities of young children. Teachers had 60 video clips that increased in difficulty over time. The video to be shown each day was pre-planned, for example “Week 1, Day 4,” but teachers could choose to redo the videos already used if they were not mastered by the whole class or most students. For example, one of the “easy” rhythmic patterns proposed at the beginning of the training was composed of 5 pulses (long, long, short, short, and long) and used only one body part, while a more complex pattern could be composed of 9 faster sounds with two alternating body parts (e.g., fingers and thighs), and could be proposed when children became more comfortable over the weeks.

The second task, “rhythmic vitamins on tape,” also involved repeating short rhythmic sequences on different body parts, but this time synchronizing with metric instrumental music. A series of 60 one-minute video clips of increasing difficulty were also provided to the teachers, and projected to the children each morning, using different musical styles (e.g., jazz, classical, and world music), different time signatures (e.g., 3/4, 2/4, 4/4, and 6/8), and different tempi (slow, medium, and fast). The addition of soundtracks allowed the children to synchronize to an external tempo while reproducing rhythms.

The third task, also one-minute in duration, involved moving in rhythm in the classroom while listening to a piece of music. Different instrumental soundtracks of various tempos, atmospheres, and styles were proposed for this musical synchronization activity.

Finally, the fourth task, which lasted 2 min, consisted of a game of moving to the beat. The task could either involve walking to the tempo of instrumental music for 2 min (with different instructions, such as imitating an animal), or inventing a movement to accompany the rhythm of the music. The task could also consist of following the tempo played by the teacher on the tambourine, who played regular fast, medium, or slow beats, without a musical soundtrack. These activities allowed for students to synchronize their body movements to a given metric. Materials for this rhythm training included an activity description guide, videos, and audio tapes.

##### Art training

For the duration of the project, the teachers had 60 digitized visual artworks (such as paintings and sculptures) according to a different theme each week (e.g., monochrome, portraits, landscapes, book illustrations, animals, graffiti, etc.). A typical art training session included five tasks for a total of 5 min each day. First, the teacher showed a reproduction of a visual artwork to children. Then, the first task was to produce, altogether, a human copy of the artwork in question. To that end, each child had to point the part of the work they wanted to reproduce, then position themself so as to represent this element in space. Each child took their turn to collectively reproduce the entire work (e.g., one child could make the beak of the eagle, the second its wings).

The second task was a static mime. The teacher asked the children, placed face to face, to choose a character or element of the artwork. They had to take its shape with their body and keep this pose for a few seconds without moving. The third task was to keep the posture chosen in the previous task and act out an emotion they felt while observing the item they chose, or an emotion they thought the object or character might feel (e.g., anger, joy, and sadness), without moving.

For the fourth task, they were asked to move through the space while keeping the emotion and character (or object) they had chosen.

Finally, the fifth task was an “imaginary object” game. The teacher mimed an object related to the artwork, which the children had to guess by miming different actions related to this object (e.g., pretending to shovel snow, make a snowball or a snowman, the object to be guessed being “snow”). Then, the children were placed in a circle, and the teacher passed the “object” to a child, who, in turn, would “mime” a movement with the object (e.g., making an angel in the snow), and then pass it to the child next to them.

If time allowed, a sixth task was proposed, where the children worked in pairs. One of them had to “draw” an excerpt from the artwork on the back of the other (with their finger). The peer then had to guess which part it was and point it out on the interactive whiteboard.

Materials for this art training included a guide to describe activities, 60 visual artworks with their description, and many suggestions of elements or emotions to exploit for each work, which did not limit the teacher and/or children in their choices. In the end, the visual arts condition was made up of activities of the same duration as the rhythm routines, so as to maintain equivalent lengths of intervention.

### Data analysis

To better understand the relationships between rhythmic, graphomotor, and literacy skills prior to the training, we first pooled the pretest data independently of the training group (since the trainings had not yet begun at the time of the pretests), and performed Pearson’s correlations analysis between all the measures, taken two by two (bivariate correlations, with appropriate Bonferroni correction, Curtin and Schulz, 1998). Next, to ensure the homogeneity of the rhythm and art groups before trainings, Student’s *t*-tests were performed between the two groups (rhythm vs. art) on the three control measures (Vocabulary, Progressive matrices, and Span tasks). Finally, to evaluate training effects, we carried out ANOVA with moment (pre vs. post-test) as the within-factor, and groups (rhythm vs. art) as the between-factors. When they were significant, interactions were decomposed with Tukey HSD *post-hoc*, and when relevant, effect sizes were also reported: Cohen’s d (Cohen, 1988) for *t*-tests and eta-squared (η^2^) for ANOVA. These analyses were realized using the JASP Team (2022) software.

## Results

### Correlations at pre-test

To investigate the relationships between rhythmic, literacy, and graphomotor skills, we first set up a correlation analysis between all the measures collected pre-test, regardless of the group (rhythm and art), for all our participants. The analyses show a significant correlation between the rhythm discrimination score and both syllable counting (*r* = 0.240, *p* = 0.036) and phoneme identification performances (*r* = 0.367, *p* = 0.001). Thus, the better the child performs on the rhythm discrimination task, the better they will perform on the syllable counting task and the phoneme identification task.

Moreover, the rhythm synchronization score selected correlates negatively with the initial phoneme identification score (*r* = −0.281, *p* = 0.014). As a reminder, a low synchronization score (Rayleigh corrected to 600 ms) reflects good rhythm synchronization performances. Thus, the more synchronized the children were to the 600 ms auditory tempo, the higher their performance in initial phoneme identification.

Finally, the rhythm synchronization score correlates negatively with the pressure exerted on the pen during writing (*r* = − 0.355, *p* = 0.002) and drawing tasks (*r* = − 0.303, *p* = 0.008). The more synchronized the children were to the 600 ms auditory tempo, the more pressure they exerted on the pen during writing and drawing tasks.

### Homogeneity of the rhythm and art groups before trainings

Results of Student’s *t*-tests (Table 2) showed no significant differences between the two groups on the three control measures (Vocabulary, *t*(74) = −0.24, ns, Progressive Matrices, *t*(74) = 0.47, ns, and Span tasks, *t*(74) = 0.19, ns), indicating that the 2 groups were well homogenized prior to trainings on these skills.

**Table 2:** Mean (*SD*) of the 3 control measures (vocabulary, progressive matrices, and span tasks) at pre-test, for the 2 groups (rhythm and art).

Control tasks	Rhythm training pre-test	Art training pre-test	Student’s *t*-test	*p*-value
Vocabulary/54	26.88 (5.05)	26.65 (2.06)	*t*(74) = −0.24	*p* = 0.81
Progressive matrices/36	18.62 (4.29)	19.09 (4.09)	*t*(74) = 0.47	*p* = 0.64
Span tasks/15	6.91 (1.55)	6.59 (1.78)	*t*(74) = 0.19	*p* = 0.85

### Training effects for rhythm measures

Before observing possible transfer effects from rhythmic learning to other non-rhythmic skills, we verified that the rhythmic interventions influenced rhythmic skills. Descriptive statistics (mean and standard deviation) for the two rhythm measures are presented in Table 3, for the pre-test and the post-test, in function of rhythm training and art training.

**Table 3:** Mean (*SD*) of the rhythm measures at pre and post-test and results from the ANOVA (*F*, *p* values and size effect) for the interaction between pre versus post-test moment and groups (rhythm vs. art).

Rhythm	Rhythm training pre-test	Rhythm training post-test	Art training pre-test	Art training post-test	*F*	*p*-value	*η* ^2^
Discrimination score	1.48 (0.63)	1.38 (0.80)	1.26 (0.74)	1.18 (0.72)	*F*_(1,74)_ = 0.07	0.93	0.00004
Synchronization score	475.86 (86.70)	428.36 (90.85)	445.07 (92.15)	467.77 (94.08)	*F*_(1,74)_ = 6.7	0.012	0.036

**Figure 1:**
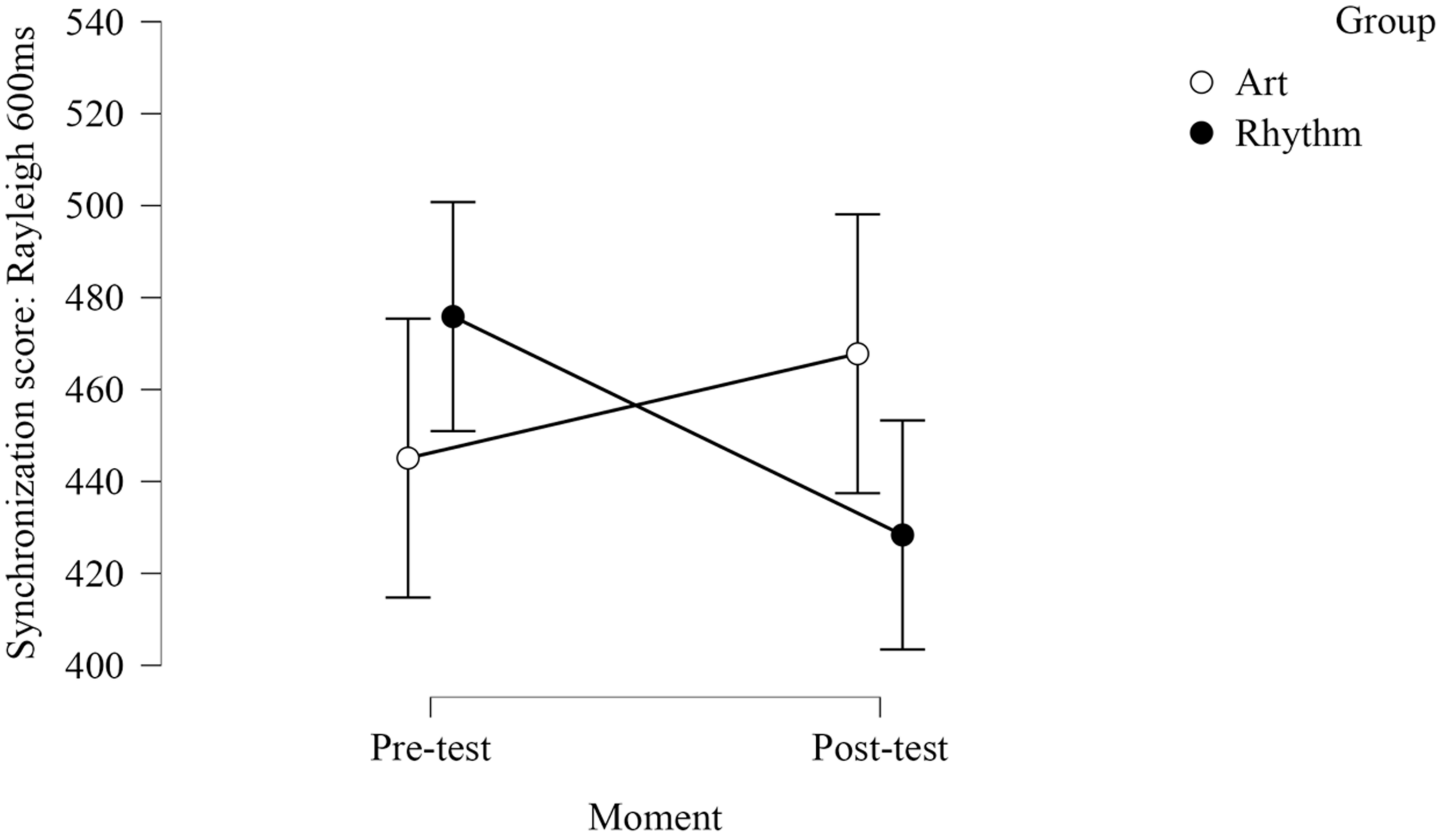
Training effect (difference between pre-test and post-test) for rhythm synchronization score (Rayleigh corrected to 600 ms) for art and rhythm groups.

Neither the rhythmic nor the visual art training improved the discrimination score, but synchronization score was significantly improved in the rhythm group (*cf.* Figure 1 below, *post-hoc* comparison: in the rhythm group: pre-test = 475.86; post-test = 428.36; *p* = 0.05).

### Training effects for literacy measures

Descriptive statistics (mean and standard deviation) of the five literacy measures are presented in Table 4 for the pre-test and the post-test in function of rhythm training and art training.

**Table 4:** Mean (*SD*) of the literacy measures at pre and post-test, and results from the ANOVA (*F*, *p* values and size effect) for the interaction between pre- versus post-test moment and groups (rhythm vs. art).

Literacy	Rhythm training pre-test	Rhythm training post-test	Art training pre-test	Art training post-test	*F*	*p*-value	*η* ^2^
Syllable counting/6	5.70 (0.59)	5.73 (0.54)	5.52 (0.65)	5.64 (0.69)	*F*_(1,74)_ = 0.2	0.61	0.001
Syllable deletion/8	2.31 (1.22)	2.56 (1.51)	2.21 (1.59)	2.12 (1.27)	*F*_(1,74)_ = 0.9	0.34	0.004
Rime discrimination/8	4.91 (1.39)	5.56 (0.96)	5.03 (1.27)	5.56 (0.83)	*F*_(1,74)_ = 0.1	0.70	0.0007
Phoneme identification/8	5.02 (2.43)	6.75 (1.57)	4.18 (2.79)	6.72 (1.76)	*F*_(1,74)_ = 2.5	0.12	0.007
Invented spelling/17	6.53 (3.01)	8.82 (3.75)	5.23 (6.68)	8.63 (3.29)	*F*_(1,74)_ = 1.8	0.18	0.009

Neither the rhythmic nor the art training improved the 5 measures of literacy.

### Training effects for graphomotricity measures

Descriptive statistics (mean and standard deviation) of the five graphomotricity measures are presented in Table 5, for the pre-test and the post-test in function of rhythm training and art training.

**Table 5:** Mean (*SD*) of the graphomotricity measures at pre- and post-test, and results from the ANOVA (*F*, *p* values and size effect) for the interaction between pre- versus post-test moment and groups (rhythm vs. art).

Grapho motricity	Rhythm training pre-test	Rhythm training post-test	Art training pre-test	Art training post-test	*F*	*p*-value	*η* ^2^
Legibility of drawing (/6)	2.02 (1.20)	1.33 (0.57)	1.97 (1.11)	1.24 (0.43)	*F*_(1,74)_ = 2.35	0.13	0.011
Speed in drawing (in cm/s)	5.37 (1.71)	5.66 (3.14)	5.77 (1.37)	5.66 (2.77)	*F*_(1,74)_ = 3.06	0.08	0.017
Pressure in drawing	18,352 (4033)	11,697 (4197)	18,311 (3839)	12,624 (4022)	*F*_(1,74)_ = 1.28	0.26	0.002
Nb. of pauses in drawing	19.15 (12.71)	5.49 (2.72)	17.4 (10.03)	6.05 (3.86)	*F*_(1,74)_ = 2.12	0.15	0.012
Duration of pauses in drawing (in ms)	26.88 (4.61)	118.65 (78.44)	26.22 (4.11)	111 (51.84)	*F*_(1,74)_ = 4.05	0.048	0.019

**Figure 2:**
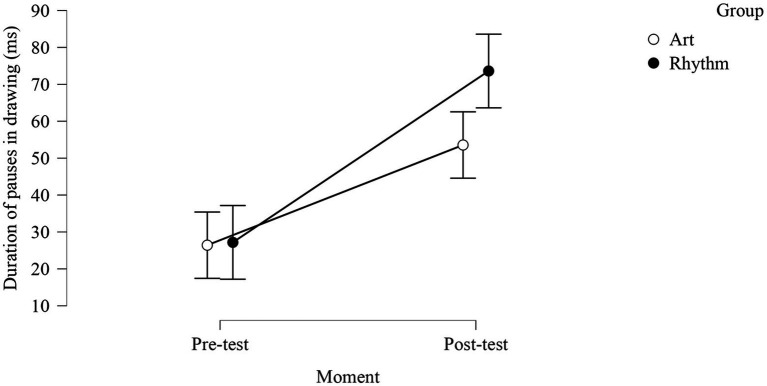
Training effect (difference between pre-test and post-test) for duration of pauses in drawing for rhythm and art training.

The results were significant only for the duration of pauses in drawing, with a significantly greater duration of pauses in the rhythm group (*cf.* Figure 2 below, *post-hoc* comparison: in the rhythm group: pre-test = 27.18, post-test = 73.61 *p* = 0.008) as compared to the art group.

## Discussion

The purpose of this research was to investigate possible links between three skill areas: rhythm, literacy, and graphomotricity. To that end, children were evaluated on several tasks measuring those skills, and we looked for correlations between them. We then engaged one-half of students in daily rhythm training, and the other in arts training. We hypothesized that the rhythmic training would improve rhythmic skills, which would allow a transfer effect to phonological and graphomotor skills.

As expected, results showed positive correlations between rhythm discrimination measures and PA, specifically syllable counting and identification of the first phoneme tasks, and between synchronization measures (Rayleigh 600 ms – 20%) and identification of first phoneme. Our results confirm that kindergartners’ rhythmic abilities were strongly related to their PA (Moritz et al., 2013). Syllables are a very important element in language segmentation, specifically in French, which is a syllable-timed language (one characterized by the recurrence of a given element at regular intervals vs. stress-timed languages, in which the vowel is accented; Wenk and Wioland, 1982). Good rhythmic skills seem to underlie the ability to break language into syllables, and as previously noted, when children are learning to read, those who can easily segment and blend sounds are able to use this knowledge to read and spell. In contrast, our results did not show any correlation with the syllable deletion task. The scores for this task are very low and this can be explained by the fact that this task also involves working memory capacities, since it consists, in addition to cutting the word into syllables, of maintaining it in memory and removing the syllable “to be deleted” in order to reconstruct the new item by merging the remaining syllables. Some studies have shown that working memory accounts for the variance in some PA subtasks, and in our case, it would appear that 5-6-year-olds are not yet able to perform the task correctly (Oakhill and Kyle, 2000). Moreover, the fact that the rhythm discrimination task is correlated with two of the PA tasks, while the synchronization task is correlated with only one, is consistent with the results indicating that the rhythm perception task could be more related to phonological skills than motor production rhythm skills (Zhang et al., 2017). In line with recent results (Bonacina et al., 2020), showing that the ability to drum to a beat predicts rapid letter-naming abilities, whereas the ability to tap rhythmic patterns predicts PA, our results also highlight that rhythm is a multi-dimensional skill, and that more studies are needed to better specify which rhythmic component (perception, reproduction, synchronization, etc.) underlies which language component (phonological processing, letter processing, etc.), and how this might change with age.

The results of these correlation analyses also show a negative correlation between the 600 ms synchronization score and pen pressure during the writing and drawing tasks. Thus, the more synchronized the children were, the more pressure they exerted on the pen. As indicated in the introduction, some correlational, behavioral, and neuroimaging studies have already suggested relationships between motor skills and rhythmic abilities (Grahn and Brett, 2007; Monier and Droit-Volet, 2019). For example, Monier and Droit-Volet (2019) assessed 57 children aged 5 to 8 years using sensorimotor synchronization and continuation tasks. For both tasks, the stability of production and the ability to produce a new tempo (different from the preferred tempo) were correlated with fine motor skills. However, not many studies have examined at pen pressure, and thus the interpretation of our result is complex. On the one hand, we could have hypothesized a correlation in the opposite direction of the one observed, that is that better rhythmic skills would lead to a decrease in pen pressure. This would build on, for example, the work of Degé et al. (2020), in which 25 preschoolers were randomly assigned to music training (including rhythmic exercises, dance, and familiarization with pitch intervals) and sports training for 20 min, 3 times per week, for 14 weeks. Children in the music group showed better motor inhibition, suggesting that music/rhythmic training may contribute to the development of motor control. On the other hand, we know that at this age, children do not have fully mature graphomotor gestures (Vaivre-Douret et al., 2021) and it is possible that those who perform better on rhythmic aspects hold their pen better, and would thus present a more fluid and more constant tracing, with perhaps more pressure to exert better control on the pen. However, this should also result in a better legibility of the writing, which is not the case in our results.

Our results also show that after only 10 weeks of rhythm training, for 5 min a day each morning, children in the rhythm group improved significantly better on the synchronization task (Rayleigh 600 ms), which is not the case in the arts group. Even if the effect size is small, to our knowledge, this is the first time that improvements of rhythm abilities have been observed following such a short training period, and for training carried out in the school by the respective teachers of each class as a daily routine (even if in reality, the duration of the trainings was 7–8 min rather than 5). Indeed, in the few studies on rhythmic training, it is sometimes surprising to find that rhythmic skills were not measured (neither before nor after learning), but only trained, which limits the scope of the results and the interpretation of possible transfer effects to other skills (e.g., Patscheke et al., 2018). Moreover, trainings are usually performed by external professionals, such as researchers, research assistants, or musicians. While this perhaps guarantees a better implementation of the training program, it is also more expensive and limits the development of this kind of practice. In our case, we requested that the teachers realize the trainings themselves. The fact that the trainings took place in real everyday school situations increases the ecological validity of the research (Laurencelle, 2005). This significant improvement may also be related to the fact that the trainings involved the whole body, and not only the motor response of the hand, as is often the case in other studies. This finding falls within the field of embodied cognition (Barsalou, 2008; Kiefer and Trumpp, 2012) and corroborates the growing research showing the importance of the use of the whole body in learning. In education, musical practice and rhythmic activities are considered “multimodal” because they require the processing of visual, auditory, and motor information (Dormoy, 2019).

Nevertheless, our results do not show any specific improvement in the rhythmic group for either phonological awareness or graphomotor skills, except for an increase in the duration of pauses while drawing in the rhythm group. This result is probably related to the improvement in the Rayleigh score corrected to 600 ms in this rhythm group. Indeed, it can be assumed that the improvement in the prediction process brought about by the rhythmic training will be reflected in the ability to inhibit a motor behavior. The increase in the duration of pauses can be interpreted as a more controlled and better anticipated behavior, allowing for better preparation of the graphomotor gesture (Frischen et al., 2022).

Therefore, with the exception of this last small result, our study does not allow us to form a conclusion about transfer effects between rhythmic skills and other non-rhythmic/general skills. Several explanations can be offered to explain this lack of effect. First, as explained above, most studies showing links between rhythmic skills and other skills use only correlational and/or mediation analysis. In interventional studies involving rhythm, the results are mixed: some of them demonstrated positive impact of rhythmic training on phonological processing and reading (e.g., Huss et al., 2011; Rautenberg, 2015; Ozernov-Palchik et al., 2018), but not all studies have found such relations (Anvari et al., 2002). Thus, Gordon et al. (2015) demonstrated that the association between PA and rhythm in 6-year-olds was no longer significant after non-verbal IQ was partialled out. Patscheke et al. (2018) specifically trained 4 to 6-year-olds on either pitch or rhythm, and their results show that after the training phase, only the pitch program showed a positive effect on PA. Overall, results of longitudinal studies are not so consistent, and probably depend on many factors, such as the duration and content of the training, the tests used, and the age of the children. On this last point, we had chosen kindergarteners, for whom it seems that rhythmic training interventions are the most effective (Hierbert and Taylor 2000; Gordon et al., 2015; Ozernov-Palchik et al., 2018). Moreover, in some of these studies, the interventions involved both non-speech rhythm tasks (e.g., playing a musical instrument, hand clapping) and speech rhythm tasks (e.g., segmenting a word into syllables by hand clapping; Lê et al., 2020), whereas in our study, we only trained motor rhythmic skills. This may also explain in part why we did not observe significant improvement in literacy skills especially due to the rhythm training. Finally, the relationship between rhythm and literacy development has been repeatedly observed in children with learning disabilities (Wolff, 2002; Thomson and Goswami, 2008; Corriveau and Goswami, 2009; Huss et al., 2011; Habib et al., 2016; Lundetræ and Thomson, 2018), but only a handful of studies have been conducted in typically developing children (David et al., 2007; Zhang et al., 2017; Ozernov-Palchik et al., 2018). It is thus well-known that children with developmental dyslexia show an impairment of temporal processing; for example, they show greater variability when asked to tap along a metronome (Thomson and Goswami, 2008) and difficulties reproducing patterned rhythms of tones. Many studies showed a strong link between rhythm skills and phonological and reading abilities in this population, and a possible improvement of the latter skills through rhythmic and/or musical training. The lack of a transfer effect in our case could therefore be explained by a specific influence of rhythm on phonological skills in children with learning disabilities and/or a low level of PA, which was not the case for our study population.

In conclusion, our research shows that teachers can take advantage of a rhythmic learning program and introduce it into their daily routine in order to improve the rhythmic skills of their students. Even if our results did not show any particular improvement in non-rhythmic skills, such as phonological awareness or graphomotricity after this learning, other studies have been able to show such improvements, which may require longer training times or a focus on children struggling with language-based deficits such as developmental dyslexia to be seen. Our research thus indicates avenues for educational practice, in terms of non-verbal (rhythmic) intervention as a way to boost phonological awareness in children who struggle with reading.

## Data availability statement

The raw data supporting the conclusions of this article will be made available by the authors, without undue reservation.

## Ethics statement

The study was reviewed and approved by UQAC Human Research Ethics Committee (#602.463.05) which follows the framework of Enoncé de politique des trois conseils: éthique de la recherche avec des êtres humains. Written informed consent to participate in this study was provided by the participants’ legal guardian/next of kin.

## Author contributions

AF, AL, and LP contributed to conception and design of the study. IC, JP, and LP performed the statistical analysis. AF wrote the first draft of the manuscript. All authors contributed to manuscript revision and read and approved the submitted version.

## Funding

This research was supported by the program of development and support to research and creation of the University of Quebec in Chicoutimi, as well as by the Mission Recherche at the University Paris-Est Créteil Val-de-Marne.

## Conflict of interest

The authors declare that the research was conducted in the absence of any commercial or financial relationships that could be construed as a potential conflict of interest.

## Publisher’s note

All claims expressed in this article are solely those of the authors and do not necessarily represent those of their affiliated organizations, or those of the publisher, the editors and the reviewers. Any product that may be evaluated in this article, or claim that may be made by its manufacturer, is not guaranteed or endorsed by the publisher.

## Footnotes

^1^All these unpublished copying tasks were developed in the framework of the ANR-FRQSC NUMEC project (leaders: M.-F. Morin and D. Alamargot).
